# Severe allergic reactions to guinea pig

**DOI:** 10.1186/1476-7961-3-14

**Published:** 2005-10-27

**Authors:** Michael C Zacharisen, Michael B Levy, Jeffrey L Shaw, Viswanath P Kurup

**Affiliations:** 1 Section of Allergy/Immunology, Department of Pediatrics, Medical College of Wisconsin, Milwaukee, Wisconsin, USA; 2 University Pediatrics and Family Allergy, Huntington, West Virginia, USA; 3 Zablocki VA Medical Research Center, Milwaukee, Wisconsin, USA

**Keywords:** guinea pig, allergy

## Abstract

**Background:**

Allergic sensitization and reactions to guinea pig (*Cavia porcellus*) have been well documented in laboratory animal handlers, primarily manifesting as rhinitis, conjunctivitis, and asthma. Severe allergic reactions, however, are rare.

**Methods:**

We report two patients with severe allergic reactions following non-occupational exposure to guinea pigs. The first patient, an 11-year-old female, developed ocular, nasal, skin and laryngeal edema symptoms immediately after handling a guinea pig. The second patient, a 24-year-old female, developed symptoms of isolated laryngeal edema after cleaning a guinea pig cage. Percutaneous skin testing, RAST, ELISA and ELISA inhibition testing with guinea pig extract were performed.

**Results:**

Both patients had IgE-mediated allergy to guinea pig confirmed by ELISA and either RAST or skin testing. ELISA inhibition studies confirmed the specificity of the IgE reactivity to guinea pig.

**Conclusion:**

Severe IgE-mediated reactions can occur following non-occupational guinea pig exposure. Physicians should be aware of this possibility.

## Introduction

Guinea pigs are popular household pets and also used in laboratory research. Allergic symptoms including rhinitis, conjunctivitis, and asthma have been documented in laboratory animal workers exposed to guinea pigs [1-5]. An extensive review of the literature revealed no reports of severe allergic reactions resulting from guinea pig exposure. We report two patients with severe allergic reactions following direct exposure to guinea pigs in domestic settings.

## Case Reports

### Case One

An 11-year-old female with a history of migraine headaches and exercise-induced asthma (EIA) was evaluated in the Allergy clinic two months after experiencing symptoms while holding a guinea pig at her hairdresser's home. This was the only episode of symptoms associated with guinea pig exposure; she had handled the pet previously without exhibiting symptoms. Within minutes of holding the guinea pig, she developed ocular itching, lacrimation, and periorbital angioedema. Symptoms rapidly progressed to facial urticaria and angioedema, rhinorrhea, throat tightness, and dyspnea. She had difficulty speaking, repeatedly attempted to clear her throat, and expressed feelings of impending doom. There was no coughing or audible wheezing.

Treatment within 20 minutes included diphenhydramine 25 mg, followed by nebulized albuterol at an urgent care clinic. Although her vital signs were not available, her mother denied she had low blood pressure. The symptoms resolved within two hours without recurrence. Epinephrine and corticosteroids were not administered.

She has since avoided guinea pig exposure and has had no further symptoms other than those related to EIA. She reported previous casual exposure to guinea pigs without adverse reaction, but had not kept any rodents as pets. She denied symptoms on previous exposures to dogs, cats, and other caged rodents.

Her past medical history was significant for episodic bronchitis, croup, sinusitis, and migraine headaches. She had no history of perennial or seasonal rhinitis. Her only medication was propranolol for migraine prophylaxis. Prior and current spirometry was normal. An exercise challenge within the past year was consistent with EIA. Physical examination, at the time of the evaluation, was significant for allergic shiners and pale, swollen inferior nasal turbinates. Her lungs were clear, and the remainder of her examination was normal.

### Case Two

A 24-year-old female smoker with allergic rhinitis, EIA, and known cat-induced rhinitis was evaluated for a several year history of perennial rhinitis and conjunctivitis. She also described an episode of severe allergic symptoms resulting from guinea pig exposure. Within minutes of cleaning her pet guinea pig's cage, she developed throat tightness, severe dyspnea, and anxious feelings. She denied coughing, wheezing, and urticaria. A feeling of "impending doom" was not specifically stated. Her symptoms resolved spontaneously one hour after departing outdoors. She did not take medication or seek medical attention.

Her past medical history was significant for irritable bowel syndrome and gastroesophageal reflux disease. Daily medication included sertraline, nasal fluticasone, and oral contraceptives. Physical examination was significant for bilateral serous otitis media and edematous nasal turbinates. Her lungs were clear, and the remainder of her examination was normal. Spirometry was equivocal due to submaximal effort.

## Methods

### Case One

Commercial radioallergosorbent testing (RAST) [Quest Diagnostics, San Juan Capistrano, CA] was performed to a variety of animal and environmental antigens. Animal antigens included dog, cat, cow, gerbil, goat, hamster, horse, mouse, rabbit, rat, sheep, swine, and guinea pig. Environmental allergens included common pollens and molds, as well as dust mites. Percutaneous skin testing was not performed due to concurrent usage of beta-blocker medication.

### Case Two

Percutaneous skin testing with a variety of environmental allergen extracts (Greer Labs, Inc., Lenoir, NC), including cat, dog, and guinea pig antigens, was performed using DermaPIK (Greer Labs, Inc., Lenoir, NC). Histamine and albumin-saline controls were included. Commercial RAST testing was not performed.

### Both Cases

Sera from both patients and three non-atopic, adult controls were assayed for specific IgE to a freshly prepared extract from the fur of a guinea pig by enzyme-linked immunosorbent assay (ELISA) as previously described [6]. The guinea pig antigen was prepared by extracting 46 mg of fur with 7.5 ml of sterile phosphate buffered saline (PBS) incubated overnight at 4°C. The extract demonstrated 40 μg/ml of protein by bis-cinchoninic acid (BCA) protein assay. ELISA inhibition testing was performed as follows. 100 μl of sera (1:20) in PBS and 5% milk was incubated with 50 μl of several concentrations of guinea pig extract for one hour at room temperature and overnight at 4°C. Immulon II polystyrene microtiter plates (Fisher Scientific, Itasca, IL) were coated with the antigen by incubating for 2 hours at room temperature and overnight at 4°C with a 5 μg/ml dilution of the extract. The plates were then washed and blocked for one hour with PBS and 0.3 % Tween 20. Sera inhibition was continued for another hour at room temperature, then 50 μl of PBS and 0.3% Tween 20 was added to the sera to prepare for ELISA. Following washing of the plates, the sera were added to the wells and incubated for 3 hours at room temperature. Plates were washed and a 1:500 dilution of biotinylated-goat anti-human IgE was added for 1 hour. Following a 1-hour incubation with 1:1000 streptavidin-labeled peroxidase, *o*-phenylenediamine was added for 30 minutes, and the reaction stopped with 6N sulfuric acid. The optical density (OD) was read by spectrophotometry at 490 nm.

## Results

### Case One

RAST to guinea pig was strongly positive (>17.5 kU/L). All other antigens tested were negative (<35 kU/L). Complete blood count was normal. Serum IgE was 64 kU/L (<114 kU/L).

### Case Two

Percutaneous skin testing was positive (equivalent to histamine control with negative saline control) to guinea pig epithelium extract. Skin reactivity was also detected to cat dander as well as ragweed, grass, and tree pollens.

### Both Cases

**Figure 1:**
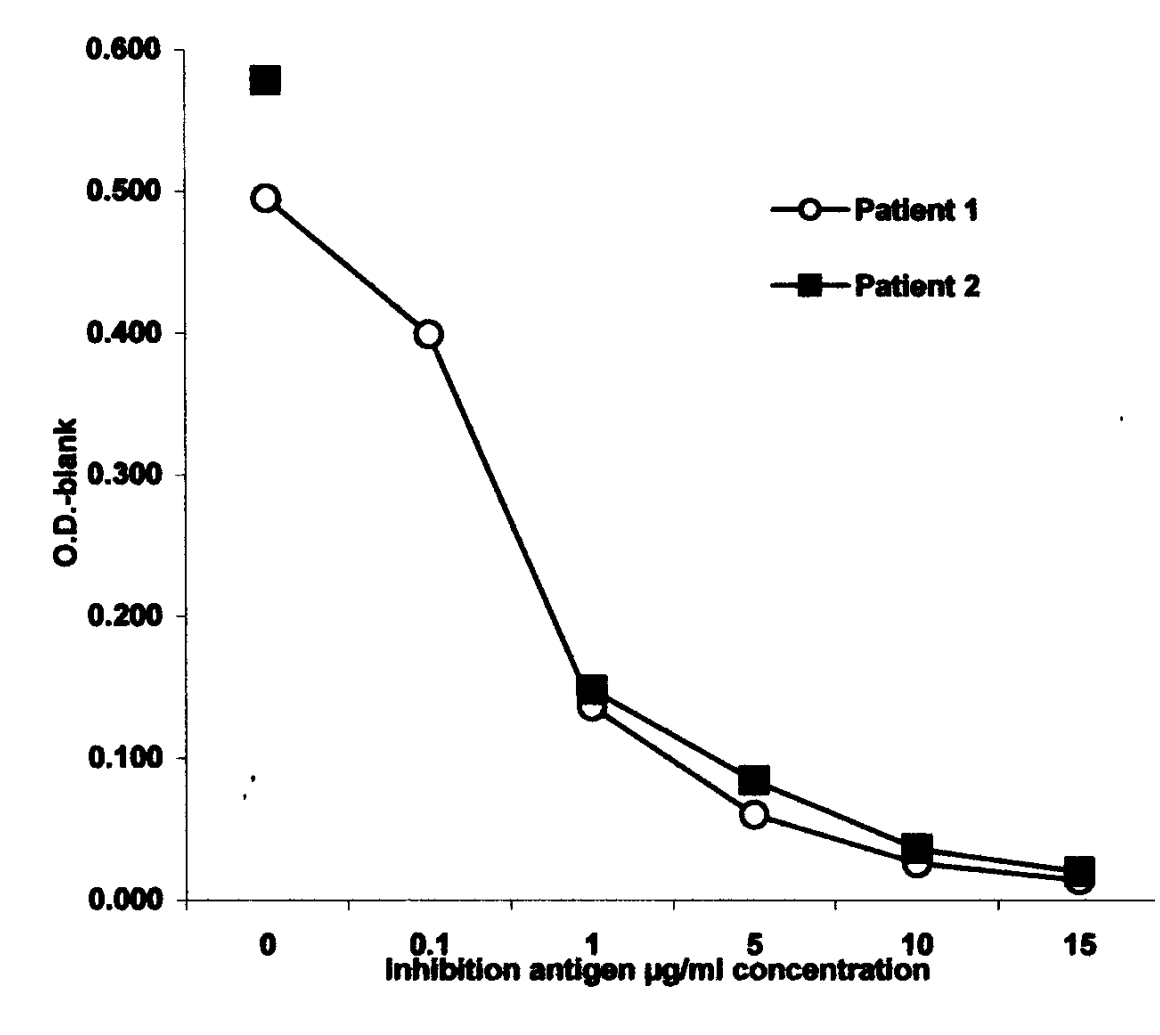
ELISA inhibition assay with guinea pig allergen resulted in complete absorption of specific IgE antibody in both patients.

ELISA demonstrated elevated levels of serum-specific IgE to crude extracts of guinea pig fur in both patients with net optical density of 1.08 and 1.29 for case 1 and 2, respectively. There was no serum-specific IgE identified in the control sera. ELISA inhibition with guinea pig allergen resulted in complete absorption of specific IgE antibody (Figure 1). The results indicate that there is no cross reactivity between hamster and guinea pig and that the antibody detected in the two cases are antigen-specific and therefore relevant. There also was minimal inhibition with hamster extract.

## Discussion

Guinea pigs are popular household pets because of their small size and the minimal time and expense involved in their care. Two major guinea pig allergens, Cav p I and Cav p II, have been identified [7,8]. Guinea pig dust, dander, fur, urine and saliva have been found to be the more potent extracts when compared to whole pelt, feces, and serum [9]. Inhalant allergens may be derived from material shed from the guinea pig coat after contamination with saliva and urine [8]. The size of airborne particles derived from guinea pig urine and dander resulting in the most allergenic activity have been shown to be of a diameter either greater than 5 microns or less than 0.8 microns, thus small enough to penetrate the lower respiratory tract when inhaled [10]. Therefore, it is not surprising that asthma can occur when sensitized individuals are exposed to guinea pigs.

In contrast to domestic settings, laboratory animal allergy (LAA) is well documented [1-5]. Approximately one third of laboratory animal workers have occupational allergy to animal dander [3]. While rats and mice are primarily used in the laboratory setting, guinea pig use is also common [4]. In a large epidemiologic study of LAA utilizing a questionnaire, the subjects handling guinea pigs reported the highest prevalence of symptoms suggestive of LAA (31%) [5].

We have described two cases of severe allergic reactions following direct contact with guinea pigs in a domestic setting. These cases are unique because they occurred with non-occupational exposure. Both sensitization and subsequent exposures were limited to domestic environments. Both patients experienced dyspnea and symptoms consistent with laryngeal edema immediately after direct contact with a guinea pig. One patient had feelings of impending doom. ELISA confirmed elevated levels of serum-specific IgE to guinea pig. Both patients demonstrated IgE inhibition with guinea pig fur extract and minimally with hamster fur extract, confirming the specificity.

Patients experiencing severe allergic reactions should be treated initially with epinephrine followed by antihistamines and corticosteroids. Although epinephrine was not administered in these cases, fortunately both patients recovered uneventfully. Primary preventative treatment should include avoidance of the offending allergen, although strong emotional attachments to pets may make adherence to this recommendation difficult. Both patients are actively avoiding contact. In addition, patients should be prescribed self-injectable epinephrine and oral antihistamines in case of accidental exposure, and be supplied with information for obtaining a medical information bracelet. Beta-blocker medications should also be avoided if possible. The patient taking propranolol for migraine headache prophylaxis was prescribed an alternative medication.

While exposure to guinea pigs rarely causes severe allergic reactions, their presence in homes, schools, and laboratories underscores the need for physicians to be aware of this possibility.

**Table 1:** 

**Test**	**Patient 1**	**Patient 2**
CBC	Normal	ND
Total serum IgE	64 lU/L	ND
Guinea pig RAST*	> 17.5 kU/L	ND
Environmental allergen RAST*	Negative	ND
Guinea pig ELISA¶	Positive at 1.08	Positive at 1.29
Percutaneous skin test		
Guinea pig	ND	Positive
Environmental allergens	ND	Positive#

* Commercial RAST

¶ In-house ELISA

ND Not done
